# Incidence and predictors of attrition from antiretroviral care among adults in a rural HIV clinic in Coastal Kenya: a retrospective cohort study

**DOI:** 10.1186/s12889-015-1814-2

**Published:** 2015-05-10

**Authors:** Amin S Hassan, Shalton M Mwaringa, Kennedy K Ndirangu, Eduard J Sanders, Tobias F Rinke de Wit, James A Berkley

**Affiliations:** KEMRI/Wellcome Trust Research Programme, Kilifi, Kenya; Kilifi District Hospital, Kilifi, Kenya; Centre for Clinical Vaccinology & Tropical Medicine, University of Oxford, Oxford, UK; Amsterdam Institute for Global health and Development, Department of Global Health, Academic Medical Center, University of Amsterdam, Amsterdam, Netherlands

**Keywords:** HIV, Antiretroviral therapy, Attrition, Lost to follow up, Mortality

## Abstract

**Background:**

Scale up of antiretroviral therapy (ART) has led to substantial declines in HIV related morbidity and mortality. However, attrition from ART care remains a major public health concern and has been identified as one of the key reportable indicators in assessing the success of ART programs. This study describes the incidence and predictors of attrition among adults initiating ART in a rural HIV clinic in Coastal Kenya.

**Methods:**

A retrospective cohort study design was used. Adults (≥15 years) initiated ART between January 2008 and December 2010 were followed up for two years. Attrition was defined as individuals who were either reported dead or lost to follow up (LFU, ≥ 180 days late since the last clinic visit). Kaplan Meier survival probabilities and Weibull baseline hazard regression analyses were used to model the incidence and predictors of time to attrition.

**Results:**

Of the 928 eligible participants, 308 (33.2% [95% CI, 30.2 – 36.3]) underwent attrition at an incident rate of 23.1 (95% CI, 20.6 – 25.8)/100 pyo. Attrition at 6 and 12 months was 18.4% (95% CI, 16.0 – 21.1) and 23.2% (95% CI, 19.9 – 25.3) respectively. Gender (male vs. female, adjusted hazard ratio [95% CI], p-value: 1.5 [1.1 – 2.0], p = 0.014), age (15 – 24 vs. ≥ 45 years, 2.2 [1.3 – 3.7], p = 0.034) and baseline CD4 T-cell count (100 – 350 cells/uL vs. <100 cells/uL, 0.5 [0.3 – 0.7], p = 0.002) were independent predictors of time to attrition.

**Conclusions:**

A third of individuals initiating ART were either reported dead or LFU during two years of care, with more than a half of these occurring within six months of treatment initiation. Practical and sustainable biomedical interventions and psychosocial support systems are warranted to improve ART retention in this setting.

## Background

The number of HIV infected individuals receiving antiretroviral therapy (ART) in Africa rose from less than a million in 2005 to more than 7 million in 2012 [1], resulting to substantial declines in HIV related morbidity and mortality [2-4]. However, attrition from ART care remains a major public health concern [5]. Indeed, the World Health Organization (WHO) has identified retention (or attrition) as one of the key reportable indicators in assessing the success of ART programs [6].

A systematic review of data from ART programs in sub-Saharan Africa (sSA) report attrition rates of 23% at 12 months, 25% at 24 months to 30% at 36 months [7], with most attrition occurring within the first year after ART initiation. The main components of attrition have been reported as loss to follow up (LFU, 56% to 59%) and death (around 40%) [5,7].

Several other studies, including a meta-analysis of data from Low- and Middle- Income Countries, have reported low baseline CD4 T-cell lymphocyte count, low baseline body mass index (BMI), advanced WHO clinical staging, younger age and male gender as independent predictors of LFU and death in ART programs [8-12].

In Kenya, the prevalence of HIV infection among adults aged 15–64 years was estimated at 5.6% in 2012 [13], with an estimated 1.6 million individuals living with HIV infection by the end of 2011 [14]. Kenya is one of the ten sub-Saharan countries that have achieved more than 60% treatment coverage [15], with an estimated 72% coverage by the end of 2011 [14]. The number of HIV-infected individuals on ART in the country has increased from an estimated 10,000 in 2003 to more than 400,000 in 2011 [14]. However, as in many other sub-Saharan countries, attrition remains one of the key challenges to the success of the national ART program.

A handful of studies have been done to describe attrition in individuals on ART in Kenya. Data from an urban slum in Nairobi report attrition probabilities of around 17% at 6 months to 35% at 24 months [16]. Follow up data from the same program report male gender, younger age and advanced HIV disease as risk factors for ART attrition [17]. More data looking at ART treatment costs from three rural outpatient clinics in the Rift Valley province report 12 months attrition of between 16% and 20% [18].

To continuously evaluate the success of ART programs in sSA, data on attrition as one of the key WHO reportable indicators should be periodically reported. To date, there is a paucity of data to describe attrition from ART care in Coastal Kenya. This study aimed to contribute to this pool of data by describing the incidence and predictors of attrition among adults initiating ART in a rural HIV clinic in Coastal Kenya.

## Methods

### Study site

The study was carried out at the HIV clinic in Kilifi District Hospital (KDH), a secondary level public health facility located in a rural part of Coastal Kenya and with a catchment population of an estimated 260,000 people [19]. The clinic began providing HIV services in 2004 and according to the Kenyan National AIDS and STI Control Program guidelines [20,21].

During the study period, ART eligibility was based on WHO clinical staging (III or IV, regardless of CD4 T-cell count) and CD4 T-cell count (<350 cells/ul, regardless of clinical staging). Individuals meeting the eligibility criteria were taken through ART preparedness and counseling, started on a standard ART regimen and given an initial two weeks appointment to assess progress and side effects. Thereafter, monthly or two monthly follow-up appointments were given based on adherence and distance from the clinic. At the time of the study, the clinic did not have an active defaulter-tracing program, which was mostly due to resource constraints. Instead, a passive surveillance approach was used. Patients who did not return for follow up care were considered LFU. Deaths were reported by family members, friends and relatives, and documented as such.

### Study design

A retrospective cohort study design was used. HIV-infected individuals aged ≥15 years initiated ART in the clinic over a three-year period (between January 2008 and December 2010) were considered eligible. Individuals who initiated ART and were transferred-in from other facilities were excluded from the analyses. Eligible participants were followed up for a period of 2 years from the date of ART initiation.

### Sources of data

These have been described elsewhere [22]. In brief, sociodemographic data including gender, date of birth, marital status, highest level of education, religion and residence sub-location were routinely collected at the time of registration into HIV care on standardized forms by trained counselors and fieldworkers. Actual distance between individual’s sub-location and the hospital was estimated in kilometers (km) using ArcInfo (ArcCatalog v.9.2).

Clinical data including anthropometry, WHO clinical staging, ART start date, ART regimen and appointment dates were routinely captured at every clinic visit in real time on standardized forms by trained clinicians. Pre-ART duration was defined as the period from registration into HIV care to ART initiation. Laboratory results, including CD4 T-cell lymphocyte counts were also captured.

A trained data clerk entered these data into an electronic data system, which was implemented in 2007. Individuals initiated ART prior to or in 2007 were hence excluded from the analysis due to lack of follow up data there from.

### Outcome definition

The primary outcome was attrition from ART care. For the purpose of this study, attrition was defined as individuals on ART care who were either LFU or reported dead at the end of the 2 years follow-up period.

Various studies have used different definitions of LFU in the HIV context. Empiric data from more than 100 HIV treatment programs in Africa, Asia and Latin America were used to determine the best performing universal definition of LFU, and recommend use of ≥180 days since last clinic visit as standard definition of LFU in HIV programs [23]. For the purpose of this study, LFU was therefore defined as individuals who were ≥180 days late since their last clinic visit.

To facilitate survival analysis, we assumed that individuals who initiated ART but never returned over the 2 years follow up period contributed one day of follow up each. Individuals remaining in care were censored at the end of the 2 years of follow up. Individuals who were either reported dead, LFU or had transferred care to other health facilities were censored at their last clinic visit date.

### Data analysis

A distribution of baseline characteristics of the study participants by gender was done. Continuous data were presented using medians and Interquartile ranges (IQR). Categorical data were presented using frequencies and percentages. Participants with missing data were coded and presented as separate categories within variables.

Kaplan Meier (KM) survival curves were used to describe the probability of attrition from ART care over follow up time. Weibull baseline hazard regression analyses were used to model the incidence of attrition over time. Univariable Weibull regression analyses were done to assess for individual predictors of time to attrition. To ensure representativeness of the study population, and to avoid potentially excluding a biased population from the analyses, categories with >20% missing observations within variables were also included in the regression analyses. Crude Hazard Ratios (cHR), 95% CI and Likelihood Ratio Test (LRT) p-values were presented.

Multivariable Weibull regression analyses were done to determine independent predictors of time to attrition. A forward stepwise model building approach was used. Predictors with a LRT p-value of <0.05 from the univariable analyses were carried forward to the multivariable analyses. Based on literature and their potential for significance, gender, age, BMI and CD4 T-cell count were considered *a*-*priori* predictors of time to attrition and were also included in the multivariable analyses. Adjusted Hazard ratios (aHR), 95% CI and LRT p-values were presented.

All data analyses were carried out using Stata statistics package (Stata 12.0, StataCorp, College Station, Texas, USA).

### Ethical considerations

These analyses were based on data routinely collected for a surveillance project on antiretroviral drug resistance and treatment outcomes in Kilifi, Kenya. Science and Ethics approvals were granted by the Scientific Steering Committee and the National Ethics and Review Committee of the Kenya Medical Research Institute respectively (SSC No. 1341).

## Results

### Cohort characteristics

**Figure 1:**
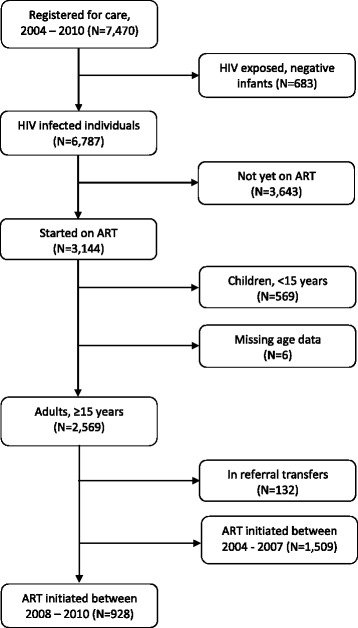
Flow diagram illustrating the eligibility of the HIV patient population to study attrition in a rural HIV clinic in Coastal Kenya between 2004 and 2010 (N = 7,470).

Overall, 7,470 individuals were registered for HIV care in the clinic between 2004 and 2010. The study cohort population comprised 928 adults initiating ART between January 2008 and December 2010 (Figure 1). Of these, 666 (71.8%) were women and 433 (46.7%) did not have a baseline CD4 T-cell count (Table 1).

**Table 1:** **Distribution of baseline characteristics in HIV-infected adults initiated antiretroviral therapy in a rural HIV clinic in Coastal Kenya (N = 928)**

**Characteristics**	**Categories**	**Male (N = 262)**	**Female (N = 666)**	**Total(N = 928)**
*Age (years)	Median	39.1	34.8	36.2
	[IQR]	[34.7 – 45.5]	[29.1 – 41.1]	[30.2 – 42.5]
Age group (years)	15 – 24	7 [2.7]	88 [13.2] 249	95 [10.2]
	25 – 34	66 [25.2]	[37.4]	315 [33.9]
	35 – 44	120 [45.8]	210 [31.5]	330 [35.6]
	≥ 45	69 [26.3]	119 [17.9]	188 [20.3]
Marital status	Single	19 [7.3]	50 [7.5]	69 [7.4]
	Married, Monogamous	169 [64.5]	229 [34.4]	398 [42.9]
	Married, Polygamous	30 [11.5]	126 [18.9]	156 [16.8]
	Separated/Divorced/Widowed	44 [16.8]	259 [38.9]	303 [32.7]
	Missing	0 [0.0]	2 [0.3]	2 [0.2]
Religion	Christian	168 [64.1]	399 [59.9]	567 [61.1]
	Muslim	45 [17.2]	114 [17.1]	159 [17.1]
	Others	46 [17.6]	146 [21.9]	192 [20.7]
	Missing	3 [1.2]	7 [1.1]	10 [1.1]
Education	No formal education	30 [11.5]	285 [42.8]	315 [33.9]
	Primary education	139 [53.1]	281 [42.2]	420 [45.3]
	Secondary/Higher education	90 [34.4]	94 [14.1]	184 [19.8]
	Missing	3 [1.2]	6 [0.9]	9 [1.0]
*Distance from hospital (km)	Median	7.8	7.8	7.8
	[IQR]	[2.2 – 16.8]	[2.2 – 17.7]	[2.2 – 17.7]
Distance from hospital (km)	0 – 5	96 [36.6]	251 [37.7]	347 [37.4]
	5 – 10	54 [20.6]	148 [22.2]	202 [21.8]
	≥ 10	75 [28.6]	189 [28.4]	264 [28.5]
	Missing	37 [14.1]	78 [11.7]	115 [12.4]
*Pre-ART duration (months)	Median	4.7	6.6	6.3
	[IQR]	[1.6 – 16.7]	[2.2 – 23.3]	[1.9 – 21.4]
Pre-ART duration groups (months)	0 – 12	184 [70.2]	420 [63.1]	604 [65.1]
	12 – 36	58 [22.1]	166 [24.9]	224 [24.1]
	≥ 36	20 [7.6]	80 [12.0]	100 [10.8]
Baseline WHO clinical staging	Stage I/II	118 [45.0]	393 [59.0]	511 [55.1]
	Stage III/IV	120 [45.8]	238 [35.7]	358 [38.6]
	Missing	24 [9.2]	35 [5.3]	59 [6.4]
*Baseline BMI (Kg/m^2^)	Median	19.1	19.3	19.3
	[IQR]	[17.3 – 21.3]	[17.3 – 21.9]	[17.3 – 21.6]
Baseline BMI groups (Kg/m^2^)	<16.0	30 [11.5]	67 [10.1]	97 [10.5]
	16.0 – 18.5	62 [23.7]	172 [25.8]	234 [25.2]
	>18.5	127 [48.5]	347 [52.1]	474 [51.1]
	Missing	43 [16.4]	80 [12.0]	123 [13.3]
*Baseline CD4 (cells/ul)	Median	135	166	157
	[IQR]	[30–213]	[53–240]	[46–234]
Baseline CD4 groups (cells/uL)	0 – 100	64 [24.4]	121 [18.2]	185 [19.9]
	100 – 350	68 [25.9]	197 [29.6]	265 [28.6]
	>350	11 [4.2]	34 [5.1]	45 [4.9]
	Missing	119 [45.4]	314 [47.2]	433 [46.7]

ART (Antiretroviral therapy), BMI (Body Mass Index), IQR (Interquartile range), WHO (World Health Organization).

### Incidence of attrition

Of the 928 adults initiated ART and followed up for 2 years, 523 (56.4%) were retained and on active follow up while 97 (10.5%) were formally transferred to other health facilities of their choice for follow up ART care. Fifty-five (5.9%) were reported dead and 253 (27.3%) were LFU.

**Figure 2:**
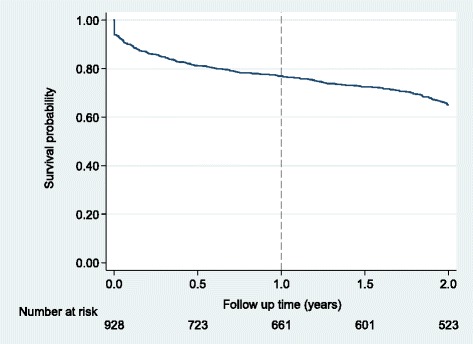
Kaplan Meier survival estimates for attrition among individuals who initiated antiretroviral therapy in a rural HIV clinic in Coastal Kenya (N = 928).

Overall, the 928 adults on ART contributed a total of 1,336 person years of observation (pyo). Of these, 308 (33.2% [95% CI, 30.2 – 36.3]) underwent attrition at an incident rate of 23.1 (95% CI, 20.6 – 25.8)/100 pyo (Figure 2). The number of individuals who had undergone attrition by 6 and 12 months were 171 (18.4% [95% CI, 16.0 – 21.1]) and 209 (23.2% [95% CI, 19.9 – 25.3]) respectively. Sixty-seven individuals (7.2% [95% CI, 5.6 – 9.1]) started treatment but did not return for follow up ART care.

### Predictors of time to attrition

In multivariable analyses, gender, baseline CD4 T-cell count and age group independently predicted attrition from ART care (Table 2).

**Table 2:** **Weibull univariable and multivariable analysis for predictors of time to attrition (lost to follow up and death) among adults initiated antiretroviral care in a rural HIV clinic in Coastal Kenya (N = 928)**

			**Univariable Regression**			**Multivariable regression (n = 761)**		
**Characteristics**	**Categories**	**Attrition, n = 308 (n/100 pyo [rate])**	**Crude HR**	**95% CI**	**LRT p-value**	**Adjusted HR**	**95% CI**	**LRT p-value**
Gender	Female	208/9.92 [21.0]	1.0	(Reference)		1.0	(Reference)	
	Male	100/3.44 [29.1]	1.3	1.1 – 1.7	0.018	1.5	1.1 – 2.0	0.014
Age group (years)	15 – 24	38/1.24 [30.6]	1.5	1.0 – 2.2		2.2	1.3 – 3.7	
	25 – 34	103/4.59 [22.4]	1.1	0.8 – 1.6		1.4	0.9 – 2.1	
	35 – 44	112/4.74 [23.6]	1.2	0.9 – 1.6		1.3	0.9 – 2.0	
	≥45	55/2.78 [19.8]	1.0	(Reference)	0.318	1.0	(Reference)	0.034
*Marital status	Single	28/0.97 [29.0]	1.0	(Reference)				
	Married, Monogamous	128/5.69 [22.5]	0.8	0.5 – 1.2				
	Married, Polygamous	48/2.39 [20.1]	0.7	0.4 – 1.1				
	Separated/Divorced/Widowed	103/4.29 [24.0]	0.8	0.5 – 1.3	0.548			
*Religion	Christian	188/8.02 [23.4]	1.0	(Reference)				
	Muslim	55/2.37 [23.3]	1.0	0.7 – 1.4				
	Others	60/2.83 [21.2]	0.9	0.7 – 1.2	0.859			
*Education	No formal education	104/4.59 [22.6]	1.0	(Reference)				
	Primary education	144/6.02 [23.9]	1.0	0.8 – 1.3				
	Secondary/Higher education	54/2.61 [20.7]	0.9	0.6 – 1.2	0.608			
*Distance from hospital (km)	0 – 5	119/5.0 [23.7]	1.0	(Reference)				
	5 – 10	57/3.2 [18.1]	0.8	0.6 – 1.1				
	≥10	82/3.9 [21.2]	0.9	0.7 – 1.2	0.270			
Pre-ART duration (months)	<12	207/8.6 [24.0]	1.0	(Reference)				
	12 – 36	68/3.2 [20.9]	0.9	0.7 – 1.2				
	≥36	33/1.5 [22.1]	0.9	0.6 – 1.4	0.663			
*Baseline WHO clinical staging	Stage I/II	141/8.13 [17.4]	1.0	(Reference)		1.0		
	Stage III/IV	124/5.05 [24.5]	1.4	1.1 – 1.8	0.009	1.1	0.8 – 1.5	0.527
*Baseline BMI (Kg/m^2^)	<16.0	41/1.12 [36.7]	2.1	1.5 – 2.9		1.6	1.0 – 2.4	
	16.0 – 18.5	72/3.53 [20.4]	1.2	0.9 – 1.6		1.0	0.7 – 1.4	
	≥18.5	128/7.58 [16.9]	1.0	(Reference)	<0.001	1.0	(Reference)	0.110
Baseline CD4 (cells/uL)	0 – 100	70/2.50 [28.0]	1.0	(Reference)		1.0	(Reference)	
	100 – 350	54/4.46 [12.1]	0.5	0.3 – 0.7		0.5	0.3 – 0.7	
	≥350	15/0.66 [22.6]	0.8	0.5 – 1.4		0.8	0.4 – 1.5	
	Missing	169/5.74 [29.4]	1.1	0.8 – 1.4	<0.001	0.7	0.5 – 1.0	0.002

ART (Antiretroviral therapy), BMI (Body Mass Index), LRT (Likelihood Ratio Test), pyo (person years of observation), WHO (World Health Organization).

*Participants excluded from Weibull regression analysis (n = 167) due to missing marital status (n = 2), religion (n = 10), education status (n = 9), distance from hospital (n = 115), baseline WHO clinical staging (n = 59) and/or BMI (n = 123) data.

**Figure 3:**
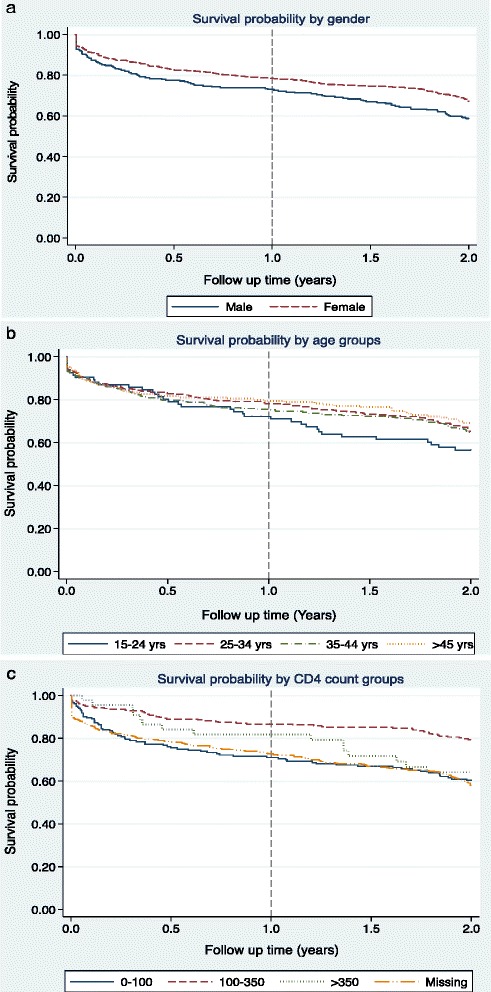
Kaplan Meier survival estimates for attrition among adults initiated ART in a rural HIV clinic in Coastal Kenya by gender **(a)**, age groups **(b)**, and CD4 T-lymphocytes count groups **(c)**.

Men had 50% higher rate of attrition from ART care compared to women (aHR [95% CI]: 1.5 [1.1 – 2.0], p = 0.014). The difference in the incidence of ART attrition between men and women was most evident within the first year of ART, with no substantial change thereafter (Figure 3(a)).

Participants with a CD4 T-cell count of 100–350 cells/ul had half the rate of attrition compared to those with a CD4 T-cell count of <100 cells/ul (0.5 [0.3 – 0.7], p = 0.002). The difference in the incidence of attrition between these two groups was highest within the first year of ART initiation, with no substantial change in the rate of attrition thereafter. Interestingly, there was no evidence for a difference in the overall attrition rates between individuals with a CD4 T-cell count of >350 cells/ul when compared to those with a CD4 T-cell count of <100 cells/ul (aHR [95% CI]: 0.8 [0.4 – 1.5]). In addition, participants with a missing baseline CD4 T-cell count had about the same incidence of attrition from ART care, compared to those with CD4 T-cell counts of <100 cells/ul. (Figure 3(c)).

After adjusting for gender, baseline WHO staging, baseline BMI and baseline CD4 T-cell count, age independently predicted attrition from ART care. Individuals aged 15–24 years were more than two-fold more likely to undergo attrition, compared to those aged ≥45 years (2.2 [1.3 – 3.7], p = 0.034). Contrary to gender and CD4 T-cell count, the variation in attrition by age groups was most evident after one year on ART, with no substantial difference in the attrition rates within the first year of treatment (Figure 3(b)).

Gender, age and baseline CD4 T-cell count positively confounded the effect of baseline WHO staging and baseline BMI on ART attrition. After adjusting for these, the effect of participants with a baseline WHO clinical stage III/IV on ART attrition was attenuated to the null, when compared to those with baseline WHO clinical stage I/II (1.1 [0.8 – 1.5], p = 0.527). Similarly, the effect of baseline BMI <16.0 was also attenuated to the null, when compared to those with a baseline BMI of ≥18.5 (1.6 [1.0 – 2.4], p = 0.110).

## Discussion

Data from a rural HIV clinic in Coastal Kenya suggest that a third of adults who initiated ART were either LFU or reported dead after 2 years, with more than a half of the attrition occurring within six months of ART initiation. These findings indicate higher levels of attrition in this setting than suggested by data from systematic reviews of ART programs across sSA reporting around 25% attrition rates after 2 years [5,7]. However, the findings are consistent with data from other parts of Kenya reporting attrition rates of about 20% at six to twelve months and 35% at 2 years [16,18]. Collectively, these findings therefore suggest that attrition remains a major challenge for ART programs in Kenya.

While literature attributes more than 40% of attrition to death [5,7], this was only 18% in our setting. Rather than suggesting a low mortality rate, we posit that this is an underestimate resulting from passive surveillance from our setting. It is likely that a higher proportion of those LFU are actually dead. Indeed, studies tracing HIV-infected LFU individuals report more than 40% deaths among those successfully traced [24,25], with the risk of mortality being highest within the first year of LFU [26,27].

At ART initiation, men were older, in more advanced stages of HIV/AIDS and had higher rates of attrition, especially within the first year of treatment. Participants with severe immunosuppression at ART initiation also had higher rates of attrition, especially within the first year of ART. These findings are consistent with data from other studies [11,12,28-30] and are suggestive of late HIV diagnosis and ART initiation, more so in men. Unlike men, women have more opportunities of being diagnosed with HIV infection and identified for ART initiation earlier on through hospital-based initiatives, including prevention of mother to child transmission interventions. This therefore underscores the importance of increasing efforts towards early identification of HIV infection in men through testing and linkage to care initiatives. In addition pre-ART lab monitoring and support systems should be strengthened for timely ART initiation [22]. Biomedical interventions, including more potent efficacious regimen, are also needed to mitigate early ART attrition through mortality in these high-risk individuals.

Younger participants had higher rates of attrition compared to the older participants. This finding has also been reported elsewhere [8,11,30]. Of interest, however, is that the difference observed in attrition rates by age group was only evident in the second year of treatment. We postulate that this may have arisen as a result of psychosocial challenges including stigma, discrimination and lack of disclosure, among the younger participants [31]. Indeed, early HIV status disclosure in adolescents on ART has been shown to improve retention [32]. Therefore, the period immediately after ART initiation offers a window of opportunity to address psychosocial challenges, including facilitating disclosure and peer support, towards mitigating later attrition from ART care among the youth and young adults.

Almost half the participants did not have a baseline CD4 T-cell count and were likely initiated ART because of advanced disease based on WHO clinical staging. This is not uncommon in resource-constrained settings and is a reflection of programmatic challenges resulting from scale up of ART, which has not always been done in tandem with the necessary lab monitoring and support systems. In addition, participants with a missing CD4 T-cell count had equally high rates of attrition as those with CD4 T-cell count of less than 100 cells/uL, suggesting that missing CD4 T-cell count at ART initiation is a good proxy for severe immunosuppression, which is associated with early mortality after treatment initiation in developing settings. In this context, therefore, it is possible that more than a half of those starting ART had severe immunosuppression.

Findings from this study should be interpreted in light of several limitations. Firstly, the passive nature of our surveillance means that the possibility of individuals who were determined LFU actually being on active care in another health facility (self-referrals) cannot be ruled out. This may have resulted in an overestimation of attrition in our analyses. However, emphasis was put on formal referrals, accompanied with a formal transfer form in all health facilities offering HIV services in this setting. All formal referrals were documented and considered in the analyses.

Secondly, and despite the passive surveillance, the presence of a research team in the clinic may have resulted to an improved quality of care, albeit in a routine programmatic context. This may have resulted in an underestimation of attrition, suggesting that attrition may actually be higher in other routine non-research facilities in rural settings in Kenya.

## Conclusions

Whilst ART scale up and treatment coverage has been impressive overall, high attrition remains a major challenge for the success of ART program in our setting. Poor pre-ART lab monitoring systems, late ART initiation, advanced HIV disease at time of ART initiation and weak support systems after treatment initiation may contribute to the high attrition rates.

Interventions aimed at identifying HIV-infected individuals and effective linkages to care, especially targeting men, coupled with strengthened periodic pre-ART lab monitoring are needed to improve timely ART initiation. In the immediate short term after ART initiation, biomedical interventions including more potent efficacious regimens should be considered to mitigate early attrition through mortality in the most immunocompromised individuals. In the long term, psychosocial support systems aimed at addressing stigma and facilitating disclosure, especially among the youth and young adults, are needed to offset late attrition.

## References

[1] UNAIDS special report: celebrating 50 years of african unity [http://www.unaids.org/en/media/unaids/contentassets/documents/unaidspublication/2013/20130521_Update_Africa.pdf]

[2] Bendavid E, Bhattacharya J (2009). The president’s emergency plan for aids relief in Africa: an evaluation of outcomes. Ann Intern Med.

[3] Jahn A, Floyd S, Crampin AC, Mwaungulu F, Mvula H, Munthali F (2008). Population-level effect of HIV on adult mortality and early evidence of reversal after introduction of antiretroviral therapy in Malawi. Lancet.

[4] World aids day report [http://www.unaids.org/sites/default/files/en/media/unaids/contentassets/documents/unaidspublication/2011/JC2216_WorldAIDSday_report_2011_en.pdf]

[5] Rosen S, Fox MP, Gill CJ (2007). Patient retention in antiretroviral therapy programs in sub-Saharan Africa: a systematic review. PLoS Med.

[6] The treatment 2.0 framework for action: catalysing the next phase of treatment, care and support. [http://whqlibdoc.who.int/publications/2011./9789241501934_eng.pdf?ua=1]

[7] Fox MP, Rosen S (2010). Patient retention in antiretroviral therapy programs up to three years on treatment in sub-Saharan Africa, 2007–2009: systematic review. Trop Med Int Health.

[8] Ekouevi DK, Balestre E, Ba-Gomis FO, Eholie SP, Maiga M, Amani-Bosse C (2010). Low retention of HIV-infected patients on antiretroviral therapy in 11 clinical centres in West Africa. Trop Med Int Health.

[9] Kouanda S, Meda IB, Nikiema L, Tiendrebeogo S, Doulougou B, Kabore I (2012). Determinants and causes of mortality in HIV-infected patients receiving antiretroviral therapy in Burkina Faso: a five-year retrospective cohort study. AIDS Care.

[10] Vella V, Govender T, Dlamini S, Taylor M, Moodley I, David V (2010). Retrospective study on the critical factors for retaining patients on antiretroviral therapy in KwaZulu-Natal, South Africa. J Acquir Immune Defic Syndr.

[11] Wandeler G, Keiser O, Pfeiffer K, Pestilli S, Fritz C, Labhardt ND (2012). Outcomes of antiretroviral treatment programs in rural Southern Africa. J Acquir Immune Defic Syndr.

[12] Gupta A, Nadkarni G, Yang WT, Chandrasekhar A, Gupte N, Bisson GP (2011). Early mortality in adults initiating antiretroviral therapy (ART) in low- and middle-income countries (LMIC): a systematic review and meta-analysis. PLoS One.

[13] Kenya AIDS Indicator Survey 2012. Preliminary Report. [http://nascop.or.ke/library/3d/PreliminaryReportforKenyaAIDSindicatorsurvey2012.pdf]

[14] Kenya AIDS Epidemic Update 2012 [http://www.nascop.or.ke/library/3d/FINALKenyaUpdat2012,30May.pdf]

[15] Regional fact sheet, Sub-Saharan Africa [http://www.unaids.org/en/media/unaids/contentassets/documents/epidemiology/2012/gr2012/2012_FS_regional_ssa_en.pdf]

[16] Unge C, Sodergard B, Ekstrom AM, Carter J, Waweru M, Ilako F (2009). Challenges for scaling up ART in a resource-limited setting: a retrospective study in Kibera, Kenya. J Acquir Immune Defic Syndr.

[17] Zachariah R, Tayler-Smith K, Manzi M, Massaquoi M, Mwagomba B, van Griensven J (2011). Retention and attrition during the preparation phase and after start of antiretroviral treatment in Thyolo, Malawi, and Kibera, Kenya: implications for programmes?. Trans R Soc Trop Med Hyg.

[18] Larson BA, Bii M, Henly-Thomas S, McCoy K, Sawe F, Shaffer D (2013). ART treatment costs and retention in care in Kenya: a cohort study in three rural outpatient clinics. J Int AIDS Soc.

[19] Scott JA, Bauni E, Moisi JC, Ojal J, Gatakaa H, Nyundo C (2012). Profile: The Kilifi Health and Demographic Surveillance System (KHDSS). Int J Epidemiol.

[20] Kenya National Clinical Manual for ART Providers [http://nascop.or.ke/library/ARTguidelines/KenyaclinicalNationalManualforARTproviders.pdf]

[21] Guidelines for antiretroviral therapy in Kenya [http://healthservices.uonbi.ac.ke/sites/default/files/centraladmin/healthservices/Kenya%20Treatment%20Guidelines%202011.pdf]

[22] Hassan AS, Fielding KL, Thuo NM, Nabwera HM, Sanders EJ, Berkley JA (2012). Early loss to follow-up of recently diagnosed HIV-infected adults from routine pre-ART care in a rural district hospital in Kenya: a cohort study. Trop Med Int Health.

[23] Chi BH, Yiannoutsos CT, Westfall AO, Newman JE, Zhou J, Cesar C (2011). Universal definition of loss to follow-up in HIV treatment programs: a statistical analysis of 111 facilities in Africa, Asia, and Latin America. PLoS Med.

[24] Weigel R, Hochgesang M, Brinkhof MW, Hosseinipour MC, Boxshall M, Mhango E (2011). Outcomes and associated risk factors of patients traced after being lost to follow-up from antiretroviral treatment in Lilongwe, Malawi. BMC Infect Dis.

[25] Yu JK, Chen SC, Wang KY, Chang CS, Makombe SD, Schouten EJ (2007). True outcomes for patients on antiretroviral therapy who are “lost to follow-up” in Malawi. Bull World Health Organ.

[26] Fox MP, Brennan A, Maskew M, MacPhail P, Sanne I (2010). Using vital registration data to update mortality among patients lost to follow-up from ART programmes: evidence from the Themba Lethu Clinic, South Africa. Trop Med Int Health.

[27] Van Cutsem G, Ford N, Hildebrand K, Goemaere E, Mathee S, Abrahams M (2011). Correcting for mortality among patients lost to follow up on antiretroviral therapy in South Africa: a cohort analysis. PLoS One.

[28] Koole O, Kalenga L, Kiumbu M, Menten J, Ryder RW, Mukumbi H (2012). Retention in a NGO supported antiretroviral program in the Democratic Republic of Congo. PLoS One.

[29] Somi G, Keogh SC, Todd J, Kilama B, Wringe A, van den Hombergh J (2012). Low mortality risk but high loss to follow-up among patients in the Tanzanian national HIV care and treatment programme. Trop Med Int Health.

[30] Weigel R, Estill J, Egger M, Harries AD, Makombe S, Tweya H (2012). Mortality and loss to follow-up in the first year of ART: Malawi national ART programme. AIDS.

[31] Rao D, Kekwaletswe TC, Hosek S, Martinez J, Rodriguez F (2007). Stigma and social barriers to medication adherence with urban youth living with HIV. AIDS Care.

[32] Arrive E, Dicko F, Amghar H, Aka AE, Dior H, Bouah B (2012). HIV status disclosure and retention in care in HIV-infected adolescents on antiretroviral therapy (ART) in West Africa. PLoS One.

